# On a New Scale for the Thermometer

**Published:** 1810-09

**Authors:** Richard Walker


### *On a new Scale for the Thermometer.*

By Mr. Richard Walker.

THERE are four different thermometric scales in use at this time.

| | | | | |
|---|---|---|---|---|
| Fahrenheit's | Freezing point of water, | 32° | Boiling point, | 212 |
| Reaumur, by De Luc, | - | 0° | - - | 80° |
| Celsius, | - - - - | 0° | - - | 100° |
| De l'Isle | - - - - | 150° | - - | 0° |

The centigrade scale now used in France, is no other than that of Celsius, to which decimal divisions have been added.

The proper place for 0, would, considering the thermometer as a measure of heat, be most properly placed at the point where heat commences; but it is probable that this point will never be ascertained, and besides the scale would be encumbered with a multiplicity of figures at the part which would be in most frequent use.

It has been ascertained by physiologists, that 62° Fahr. is that point at which the human body in a state of health is unconscious of either heat or cold, in any season of the year, and in all climates; so that any temperature above that pont, gives, under ordinary circumstances, a sensation of heat, and any temperature below it, a sensation of cold. With respect of the divisions, those of Fahrenheit seem to be the fittest, in which case, the freezing point will will be 30°, and the boiling point 150°. The only possible objection to this scale is the frequent use that it would occasion of the plus and minus characters.

For meteorological observations, this scale is peculiarly appropriated, the zero being the mean temperature between the greatest heat and the greatest cold in the hottest and coldest climates collectively, as well as in temperate climates. Thus, in Africa, the heat rises to 174° Fahr. or 112° proposed scale, and in some parts of North America the cold is 50° Fahr. or 112° pr. sc.. So also in England the heat rose to 126° Fahr. or 64° pr. sc., on the afternoon of July 13th 1808, and the cold to 2° Fahr. or 64 pr. sc. on the morning of Christmas day, 1796. The mean annual temperature of Quito in Peru, is 62° Fahr., and the limits of the variation are only 3 or 4° above or below it; and this is considered as the healthiest spot in the world. By a well known provision in the animal economy, a few degrees below 62° Fahr. is much more uncomfortable than the same difference above it.

Although Fahrenheit's division is here preferred, the mode of decimal divisions seems to be gaining ground, and might be employed; in which case, the freezing point of water would be 20°; and there would occasionally be a slight difference, but all the three leading points are constant; the remainder are not of much consequence, and the defect is common in every scale; thus quicksilver was really found to freeze at 38° and two-thirds Fahr. but is marked at 39°.

The original idea was to place 0° at the utmost degree of natural cold; and in order to ascertain this point, a table was formed from Kirwan's table of the mean annual temperature of every latitude, in which the temperature of the high latitudes are deduced by inferences drawn from the others; and if the results of this table are correct, the greatest natural cold is at 68° Fahr., or 100° below the freezing of water. If this point was adopted for the 0° of the scale, the use of the sign minus would only be required for such intense degrees of artificial cold, as were below that point, which so far as hath hitherto been produced, are only 23, namely, 91 Fahr.; the freezing point of water would be marked 100, and the boiling 280°.

